# Vulvar Hemangioma: A Review

**DOI:** 10.3390/diagnostics15101270

**Published:** 2025-05-16

**Authors:** Wing-Yu Sharon Siu, Yen-Chang Chen, Dah-Ching Ding

**Affiliations:** 1 Department of Obstetrics and Gynecology, Hualien Tzu Chi Hospital, Buddhist Tzu Chi Medical Foundation, Tzu Chi University, Hualien 970, Taiwan; wingyusharonsiu@gmail.com; 2 Division of Digital Pathology, Department of Anatomical Pathology, Hualien Tzu Chi Hospital, Buddhist Tzu Chi Medical Foundation, Hualien 970, Taiwan; s92312129@gmail.com; 3 Department of Pathology, School of Medicine, Tzu Chi University, Hualien 970, Taiwan; 4 Institute of Medical Sciences, Tzu Chi University, Hualien 970, Taiwan

**Keywords:** vulvar hemangioma, cavernous hemangioma, vascular tumor, differential diagnosis, histology

## Abstract

**Objectives**: To review the clinical presentation, diagnostic approach, treatment strategies, and outcomes of vulvar hemangiomas, and to evaluate the consistency of management practices in the absence of standardized guidelines. **Eligibility criteria**: We included case reports, case series, and observational studies describing vulvar hemangiomas with clinical, histological, and management data in human subjects. Reviews, editorials, and studies unrelated to vulvar hemangiomas were excluded. **Information sources**: Systematic searches were conducted in PubMed, Embase, Scopus, and Web of Science up to 20 February 2025, with additional citation tracking. **Results**: 85 studies were included. Data were synthesized narratively due to heterogeneity in the study design and outcome reporting. Most studies described symptomatic lesions presenting as swelling, bleeding, or pain, often misdiagnosed as Bartholin cysts or varicosities. Treatment varied widely, including observation, surgical excision, embolization, and medical therapies. No evidence-based guidelines were identified. **Conclusions**: Vulvar hemangiomas are rare and frequently misdiagnosed. While asymptomatic lesions may be observed, symptomatic cases require individualized treatment. This review highlights the need for diagnostic vigilance and the development of standardized management protocols. Further research is needed to optimize clinical outcomes.

## 1. Introduction

Vulvar hemangiomas are rare benign neoplasms that cause discomfort, sexual dysfunction, and cosmetic concerns [1]. They typically presents as purple-blue swellings on the labia majora [2]. Diagnosis often involves biopsy [2], and color Doppler ultrasound can also confirm the presence of tortuous vessels with turbulent blood flow [3]. Differential diagnoses include Bartholin cysts and vulvar varicosities. Bartholin cysts can appear macroscopically, similarly to vulvar leiomyomas, and require histopathological analysis for definitive identification [2], while vulvar varicosities may closely resemble cavernous hemangiomas, leading to potential misdiagnosis [1]. Treatment may include surgical excision, especially when symptoms progress [1,2]. However, some patients decline the intervention [2]. Spontaneous regression has also been observed in various vascular lesions. Beta-blockers or corticosteroids can be given [4,5]. The proper diagnosis and management of these rare vulvar lesions are crucial for addressing patient concerns and improving quality of life. We review vulvar hemangioma, from its symptoms to its treatment.

## 2. Methods

### 2.1. Search Strategy

PubMed, Scopus, Web of Science, and Embase were systematically searched for relevant articles published from inception to 20 February 2025. Table 1 provides an overview of the search strategy. This review followed the Preferred Reporting Items for Systematic Reviews and Meta-Analyses (PRISMA) 2020 guidelines [6].

### 2.2. Inclusion/Exclusion Criteria

The keyword was “vulvar hemangioma”. Synonyms and related terms were included to expand the scope. The references from relevant studies were also examined. The exclusion criteria were vascular lesions not located in the vulvar region, non-hemangiomatous vascular malformations (e.g., lymphangiomas, venous malformations), incomplete medical records or lack of diagnostic confirmation, patients with systemic vascular syndromes (e.g., Klippel–Trenaunay syndrome) unless the focus was strictly vulvar involvement, case reports focusing solely on vulvar varicosities or angiokeratomas, which are distinct from hemangiomas.

### 2.3. The Selection Process

The selection process involved a comprehensive review of all retrieved studies. Initially, two independent reviewers screened the titles and abstracts to assess their relevance and determine eligibility based on predefined inclusion and exclusion criteria. Studies that met the initial screening criteria or had insufficient information in the abstract were retrieved in full text for further evaluation. Any discrepancies or disagreements between the two reviewers were resolved through discussion or consulting a third reviewer to reach a consensus. This rigorous selection process ensured that only studies with an appropriate focus on vulvar hemangioma and adequate methodological quality were included in the final analysis.

## 3. Result

### 3.1. Screening Results

**Figure 1:**
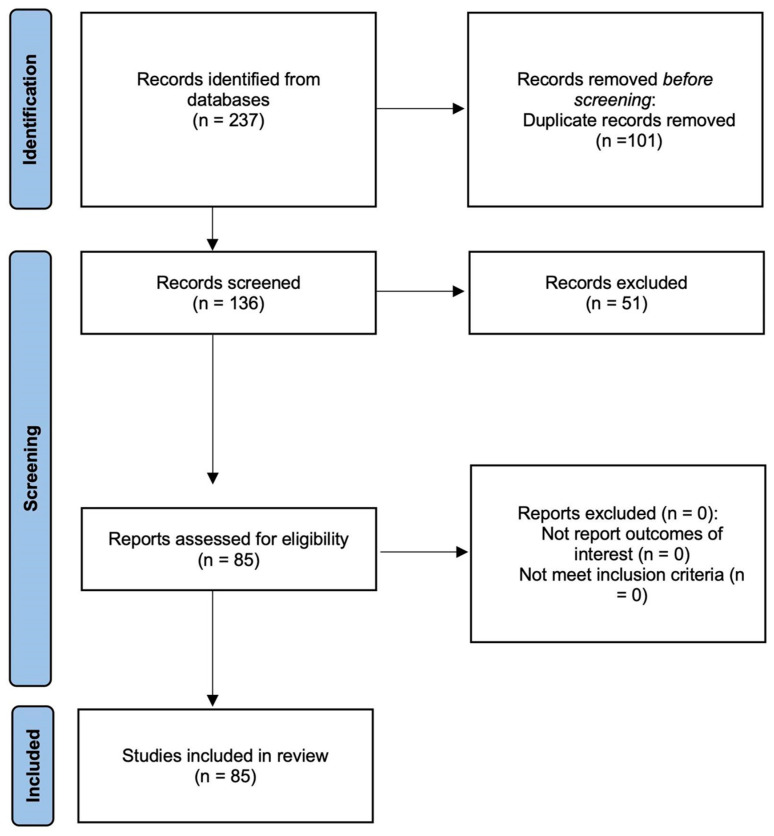
Study flow chart.

Initially, 237 articles were extracted from the databases, of which 101 were removed because of duplication. The remaining 136 articles were reviewed based on their titles and abstracts, and 51 were removed because of irrelevance. Subsequently, 85 articles were reviewed based on our exclusion criteria, and zero were excluded. Finally, 85 articles met the inclusion criteria and were included in the review (Figure 1).

### 3.2. Case Reports and Literature Review

Table 2 summarizes the reported cases of vulvar hemangiomas across different age groups, symptoms, treatments, and diagnoses.

The patient’s age ranges from 1 year to 69 years. Clinical presentations differ and include clitoromegaly, vulvar nodules, vulvar swelling, ulcerations, bleeding, pain, and discomfort.

Treatments also vary in extent, ranging from no intervention and topical therapy to surgical excision, embolization, compression bandaging, and anticoagulation therapy.

The most common pathological diagnoses are cavernous hemangioma, lobular capillary hemangioma, and vulvar hemangiomatosis. Although some patients require surgical excision owing to recurrent or symptomatic lesions, others are managed conservatively.

These cases highlight the heterogeneous clinical manifestations and management approaches for vulvar hemangiomas.

## 4. Discussion

### 4.1. Definition of Vulvar Hemangioma

Vulvar hemangioma is a rare benign vascular neoplasm that affects the female genitalia [1,2,16]. It can present as various subtypes, including cavernous, arteriovenous, and lobular capillary hemangiomas [1,7,17] (Figure 1). Owing to its rarity, no epidemiology or prevalence has been reported.

### 4.2. Clinical Significance and Impact on Quality of Life

These lesions can cause functional problems, pain, sexual dysfunction, and cosmetic concerns [1]. Women with vulvar conditions often experience a reduced quality of life, with many reporting moderate-to-extreme effects [18]. Psychological distress, including anxiety and depression, is common in affected individuals [18,19].

### 4.3. Pathophysiology

#### 4.3.1. Development of Vascular Malformations

Vascular anomalies comprise a diverse group of congenital blood vessel disorders, including tumors and malformations [20]. Hemangiomas are the most common vascular tumors, whereas lymphatic, capillary, venous, and arteriovenous malformations account for the majority of vascular malformations [20]. These lesions can affect various parts of the body, including the female genital tract [21]. Vascular malformations and hemangiomas are distinct entities with different pathogeneses and involve defective vascular remodeling in malformations [22]. Active AKT1 overexpression in endothelial cells leads to the development of cutaneous vascular malformations [23].

#### 4.3.2. Differences Between Hemangiomas and Vascular Malformations

Hemangiomas and vascular malformations are distinct entities within the spectrum of vascular anomalies. Hemangiomas are benign vascular tumors characterized by endothelial cell proliferation, whereas vascular malformations are congenital lesions arising from mesenchymal stem cell defects [24]. Differentiating these conditions is crucial for appropriate treatment. Histopathological features, such as endothelial morphology, mitotic activity, and intralesional nerve bundles, can help distinguish hemangiomas from malformations [25]. The serum levels of the vascular endothelial growth factor (VEGF) and basic fibroblast growth factor are also significantly higher in infantile hemangiomas than in vascular malformations, aiding in diagnosis [26]. The International Society for the Study of Vascular Anomalies classification provides a comprehensive framework for categorizing these lesions, including various syndromes associated with vascular malformations [27].

#### 4.3.3. Possible Genetic and Hormonal Influences

Although the pathogenesis remains unclear, genetic alterations and hormonal influences have been proposed as potential factors [28]. Some studies have suggested possible hormone dependency, particularly in relation to progesterone. A case of aggressive angiomyxoma of the vulva growing during pregnancy and strong progesterone receptor positivity has been reported [29]. Estrogen promotes the proliferation of hemangioma vascular endothelial cells in vitro [30]. Cavernous hemangiomas have also been observed to grow during puberty, further supporting the hormonal influence [30].

### 4.4. Clinical Presentation and Diagnosis

#### 4.4.1. Symptoms and Signs

Vulvar hemangiomas may appear as painful vulvar masses [17] or multiple purple-blue swellings on the labia majora [3]. Symptoms include cyclic perineal discomfort, enlargement during menses [17], and genital ulcers [2]. These lesions can cause sexual dysfunction, pain, and cosmetic concerns [1].

#### 4.4.2. Classification of Hemangiomas

The classification of hemangiomas has evolved. While earlier systems distinguish between capillary and cavernous types [31], more recent classifications include superficial, deep, mixed, and minimal or arrested growth [32]. Superficial hemangiomas are also known as capillary hemangiomas or strawberry hemangiomas [33]. These lesions are characterized by their location in the papillary (upper) dermis. This position near the skin surface likely contributes to their distinctive appearance, which may resemble strawberries; hence, the alternative name. Deep hemangiomas are also known as cavernous, hypodermal, or subcutaneous hemangiomas [32]. Unlike their superficial counterparts, these lesions are found in the reticular (lower) dermis, fat, and muscles. Their deeper locations may make them less visible on the skin surface than superficial hemangiomas. Mixed hemangiomas are combinations of superficial and deep components [32]. This type of hemangioma exhibits characteristics of both superficial and deep lesions, potentially affecting multiple layers of the skin and the underlying tissues.

#### 4.4.3. Differential Diagnosis

Differential diagnosis is crucial because these lesions can be mistaken for other conditions such as Bartholin cysts, varicosities, or melanomas [21,34]. Differential diagnoses include Bartholin cysts and vulvar varicosities. Bartholin cysts can be macroscopically similar to vulvar leiomyomas and require histopathological examination for accurate identification [2]. Meanwhile, vulvar varicosities may resemble cavernous hemangiomas [1]. Diagnosis often involves biopsy and immunohistochemistry to distinguish between endometriotic lesions and melanomas [21].

#### 4.4.4. Imaging Modalities

Imaging modalities play a crucial role in diagnosing and evaluating vulvar hemangiomas. Color Doppler ultrasonography can confirm the presence of tortuous vessels with turbulent blood flow in vulvar hemangiomas [3]. Magnetic resonance imaging (MRI) is particularly effective in evaluating the extent of these lesions, including unexpected pelvic involvement [35]. The excellent contrast resolution of MRI makes it valuable for characterizing pelvic pathology and staging vulvar malignancies [35]. Both transvaginal ultrasound and MRI are potentially useful in the preoperative assessment of vulvar masses, helping differentiate benign lesions (e.g., angiomyofibroblastoma) from more aggressive tumors [36]. These imaging techniques provide valuable information for initial evaluation, follow-up, and surgical planning of vulvar hemangiomas and their associated conditions. However, preoperative diagnosis remains challenging, and further research is needed to determine the superiority of one imaging modality over another.

#### 4.4.5. Histopathological Features

**Figure 2:**
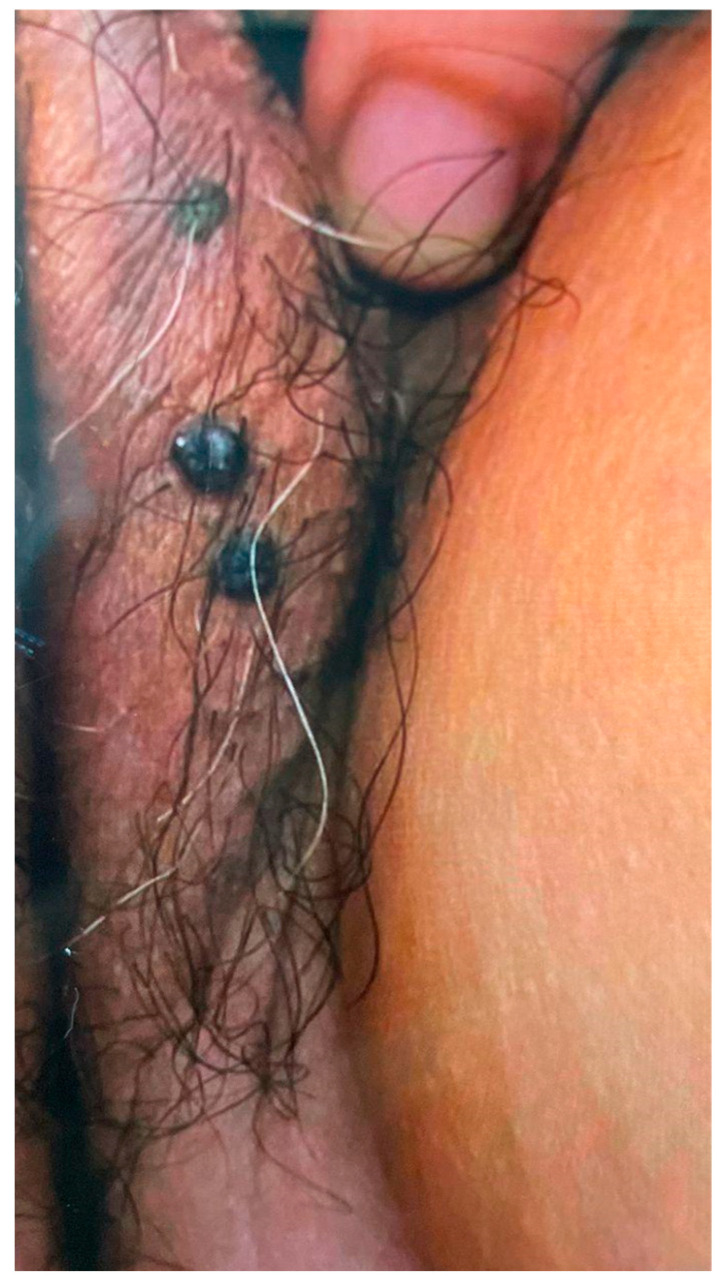
A 69-year-old female complained of vulva nodules. Gross image of the vulvar hemangioma (black nodules over the left labia majora).

**Figure 3:**
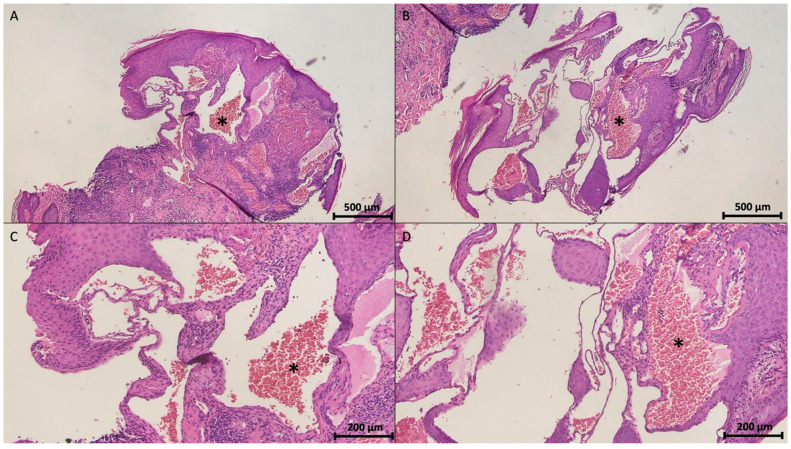
Histopathological examination of the vulvar lesion revealed a benign vascular neoplasm composed of large-caliber, blood-filled vascular channels lined by flattened endothelial cells and filled with erythrocytes (asterisks) without atypia or mitotic activity. These features are consistent with a diagnosis of cavernous hemangioma located in the vulva. ((**A**,**B**), H&E stain, 40× magnification; (**C**,**D**), H&E stain, 100× magnification).

Histologically, vulvar hemangiomas show proliferation of differently sized tortuous blood vessels within the submucosa and are closely associated with the mucosal epithelium [2] (Figure 2). The endothelial lining consists of a single layer of flattened cells, some appearing swollen but without nuclear atypia [2,37]. Larger vessels are engorged by erythrocytes, whereas smaller vessels are found in the peripheral areas (Figure 3). The superficial location of these vessels highlights their proximity to stratified squamous epithelium [2]. Additionally, an ulcerated area lacking an epithelial lining is composed of acute (polymorphonuclear cells) and chronic inflammatory cells (lymphocytes and plasma cells) along with erythrocytes, indicating an inflammatory response [2]. A rare variant, retiform hemangioendothelioma, can also occur in the vulva. Retiform hemangioendothelioma is characterized by histological features, including arborizing vascular channels in a retiform pattern. Immunohistochemical staining for CD31, CD34, and FLI-1 supports the diagnosis of a borderline-malignant tumor [38].

Comparative features and immunohistochemical profiles of mimickers such as lymphangioma, angiokeratoma, aggressive angiomyxoma, and vascular malignancies were listed in Table 3. Vulvar hemangiomas must be distinguished histologically and immunohistochemically from several mimickers, including lymphangioma, angiokeratoma, aggressive angiomyxoma, and vascular malignancies such as Kaposi sarcoma and angiosarcoma. While hemangiomas exhibit dilated, blood-filled vascular channels lined by bland endothelial cells and stain positively for CD31, CD34, and FLI-1 (but are typically GLUT-1 negative in adults), lymphangiomas show similar spaces filled with proteinaceous fluid and are D2-40 and PROX1 positive. Angiokeratomas are more superficial, with epidermal hyperkeratosis and thin-walled dermal vessels. Aggressive angiomyxomas display myxoid stroma, thick-walled vessels, and positivity for desmin, ER/PR, and variably CD34. Vascular malignancies such as Kaposi sarcoma (HHV-8+, spindle cell-rich, slit-like vessels) and angiosarcoma (atypia, mitoses, irregular channels) show cytologic atypia and infiltrative growth, which are absent in benign hemangiomas. Immunohistochemistry and architectural patterns are essential for accurate differentiation.

### 4.5. Management and Treatment Options

#### 4.5.1. Conservative Management

Although surgical excision is occasionally necessary in symptomatic cases, spontaneous regression has been observed in various types of vascular lesions. Most infantile hemangiomas (IHs) are small and undergo spontaneous regression, making them suitable candidates for conservative management [44]. Tufted angiomas, particularly congenital angiomas, show spontaneous regression during infancy and early childhood [45]. Although not specifically addressing vulvar hemangiomas, these findings suggest that observation may be possible in uncomplicated cases. The lesion without symptoms also does not need treatment [16]. The lesion was too small to cause severe symptoms, also did not need treatment like our case. These studies indicate that careful monitoring before considering invasive treatments may be appropriate for certain vascular lesions.

#### 4.5.2. Medical Therapy

##### Beta-Blockers

Beta-blockers, particularly propranolol and timolol, have emerged as effective IHs treatments. Oral propranolol is considered the first-line treatment, demonstrating superior efficacy compared to topical timolol [4]. However, topical timolol can be used as an alternative treatment for superficial IH, offering similar efficacy to the oral form but with fewer adverse effects [4,46]. Propranolol at 2 mg/kg/day is associated with better response rates than lower doses or topical timolol [4]. However, both treatments have demonstrated good to excellent responses in most patients [47]. Although generally safe, oral propranolol may cause side effects, such as somnolence, bradycardia, hypotension, and hypoglycemia [48]. The use of beta blockers for IH has significantly increased over time, with some institutions adopting outpatient treatment protocols [47,48].

##### Corticosteroids

Corticosteroids effectively treat problematic cutaneous hemangiomas, including those in the vulvar and genitourinary regions. Systemic corticosteroid therapy is associated with an 84% response rate in infants with enlarging hemangiomas [5]. The mechanism of action involves suppressing VEGF-A production by hemangioma-derived stem cells, which in turn inhibit vasculogenesis [49]. Short-term oral steroid therapy is also effective for the treatment of pediatric vaginal and urethral hemangiomas [50]. However, in some cases, corticosteroids may not be sufficient to control life-threatening complications, such as severe hemorrhage. In such cases, alternative treatments, such as selective arterial embolization followed by surgical excision, may be necessary [9]. The efficacy of corticosteroids is dose-dependent, with a mean prednisone equivalent daily dose of 2.9 mg/kg for approximately 1.8 months being effective [5].

##### Sclerotherapy

Sclerotherapy has emerged as an effective treatment for various types of hemangiomas and vascular malformations, including those in the genital area. This minimally invasive technique involves injecting sclerosing agents, such as polidocanol or sodium tetradecyl sulfate, into the affected blood vessels to induce their closure and subsequent shrinkage [51,52]. The efficacy of sclerotherapy for treating infantile hemangiomas, particularly large or pedunculated lesions, has been reported, with rapid regression and minimal complications [53]. Sclerotherapy has also been effective for the management of vaginal wall cavernous hemangiomas and vulvar varicosities in adult patients, providing symptomatic relief and reducing lesion size [52,54]. The procedure is generally well tolerated, with few reported severe complications, and often requires only one to three treatment sessions to achieve the desired results [51]. In general, sclerotherapy is a promising, cost-effective, and relatively simple treatment option for various hemangiomas and vascular malformations of the genital region.

#### 4.5.3. Surgical and Minimally Invasive Interventions

##### Laser Therapy

Laser therapy has shown promise for the treatment of various vulvar conditions. CO_2_ lasers have been successfully used to treat lymphangioma circumscriptum [55] and multiple vulvar epidermoid cysts [56]. They have also shown effectiveness for vulvar intraepithelial neoplasia and lichen sclerosus [57]. Photodynamic therapy using a red diode laser with methylene blue has been reported as a non-invasive, secure, and low-cost treatment for vulvar lymphangiomas [58]. However, although laser therapies have demonstrated efficacy in treating these conditions, evidence of their long-term outcomes and safety across different skin types is limited [57]. Most studies reported minimal short-term complications, with mild or transient pain and discomfort being the most common side effects [57]. Further long-term studies are required to establish guidelines for the optimal use of lasers in vulvar skin conditions.

##### Cryotherapy

Cryotherapy is a promising treatment modality for various vulvar vascular lesions. It has been successfully used to manage massive vulvar lymphatic leakage and lymphangiomas in cervical cancer survivors, improving the quality of life without serious complications [59]. Although surgical excision is sometimes necessary for vulvar hemangiomas [2], cryotherapy is an effective alternative. In comparing cryotherapy and electrosurgery for cherry angiomas, both methods demonstrated acceptable cosmetic results with minimal discomfort [60]. Furthermore, cryotherapy was associated with significantly better improvement in lesions with a lower recurrence rate than CO_2_ laser treatment for Klippel–Trenaunay syndrome with vascular tumors [61]. These findings support that cryotherapy may be a beneficial and less invasive treatment option for various vulvar vascular anomalies, including hemangiomas, with potential benefits concerning efficacy and recurrence rates.

##### Surgical Excision

Although some hemangiomas may regress spontaneously, symptomatic cases often require treatment [1]. Surgical excision is the primary treatment option for diagnostic confirmation and symptom relief [1,17]. In one case, a painful subcutaneous arteriovenous hemangioma was successfully treated with excision, resulting in symptom resolution at the 12-month follow-up [17]. However, some patients may decline surgical intervention despite medical advice [3].

### 4.6. Prognosis and Complications

#### 4.6.1. Natural Course and Probability of Spontaneous Resolution

The natural course of vulvar hemangiomas depends on the type and patient age. Infantile hemangiomas often follow a self-limiting pattern, grow early in life, and regress spontaneously over time [62]. However, adult cavernous and lobular capillary hemangiomas typically persist and rarely resolve spontaneously, often enlarging and causing pain, ulceration, or bleeding [13]. Spontaneous resolution is unlikely, particularly in symptomatic patients or those with progressive growth [13]. Although small asymptomatic lesions can be monitored [8], larger or symptomatic hemangiomas often require excision, embolization, or other targeted treatments [13]. Regular follow-up is essential for conservative management to assess changes in size or symptoms.

#### 4.6.2. Potential Complications

Vulvar hemangiomas can lead to several complications, depending on their size, location, and progression. Ulceration is a common complication, particularly in large or rapidly growing lesions, and often results from surface friction, trauma, or ischemia [2]. Bleeding can occur because of fragile vascular structures, and in some cases, significant hemorrhage may require medical intervention [15]. Secondary infections can develop if an ulcerated hemangioma is colonized by bacteria, leading to pain, swelling, and potential abscess formation [63]. Additionally, sexual dysfunction can arise from pain, discomfort, or psychological distress related to the presence of a lesion, affecting intimate relationships and quality of life [1]. Given these risks, timely diagnosis and appropriate management are crucial for preventing complications and ensuring optimal patient outcomes.

#### 4.6.3. Long-Term Outcomes and Recurrence Rates

The long-term outcomes of vulvar hemangiomas depend on the type, size, and treatment approach. Although infantile hemangiomas often regress spontaneously, cavernous and lobular capillary hemangiomas in adults typically persist and may require intervention if symptomatic [13]. Surgical excision has the lowest risk of recurrence; however, incomplete removal can lead to tumor regrowth. Embolization effectively reduces the lesion size, although recurrence may occur if a collateral blood supply develops [16]. Conservative management is an option for small asymptomatic lesions; however, long-term monitoring is necessary because growth or symptom development may require intervention. Although treatment outcomes are usually favorable, patients should be observed for recurrence; persistent symptoms; or late complications, such as pain, ulceration, or vascular changes [16]. Further research is needed to establish optimal treatment strategies and determine the risk of recurrence.

### 4.7. Research Gaps and Future Directions

#### 4.7.1. Need for Standardized Treatment Guidelines

Vulvar hemangiomas are rare vascular tumors with diverse clinical presentations. However, no standardized treatment guidelines exist, leading to inconsistent management strategies. Current approaches range from observation and topical treatments to surgical excision, embolization, and anticoagulation therapy, often depending on the clinician’s experience rather than evidence-based protocols. A lack of consensus on when to opt for conservative versus surgical management can result in over- or undertreatment, affecting patient outcomes. Developing standardized guidelines based on tumor characteristics, symptom severity, and potential complications is crucial for optimizing care. Management by a multidisciplinary team involving gynecologists, dermatologists, vascular surgeons, and radiologists is essential to establish clear protocols for diagnosis, monitoring, and treatment selection. Further research is needed to create evidence-based recommendations to consistently and effectively manage vulvar hemangiomas.

#### 4.7.2. Advances in Targeted Therapies

Recent studies have explored targeted therapies for hemangiomas, common tumors in infancy. Propranolol-loaded CD133 aptamer-conjugated liposome-in-microspheres have shown promise for sustained and targeted treatment, inhibiting hemangioma-derived stem cell proliferation and angiogenic factor expression [64]. Low-concentration rapamycin has also demonstrated efficacy in inhibiting hemangioma endothelial cell proliferation and migration both in vitro and in vivo by reducing the activation of the protein kinase B/mTOR/S6 signaling pathway [65]. A computational study identified 12 molecular-targeting agents with potential for restricting the proliferation and migration of hemangioma cells; these included entinostat, sorafenib, dasatinib, and sirolimus [66]. Additionally, sunitinib malate, a multi-receptor tyrosine kinase inhibitor, has shown potential in inhibiting hemangioma cell growth and migration by suppressing focal adhesion kinase signaling and reducing the expression of adhesion proteins and tumor growth factor-β1 [67]. These findings suggest a promising avenue for targeted hemangioma therapy.

#### 4.7.3. Role of Genetic and Molecular Studies in Understanding Pathogenesis

Recent genetic and molecular studies have significantly improved our understanding of hemangiomas and other vascular neoplasms. Cherry hemangiomas, the most common adult hemangiomas, frequently harbor mutations in the GNA14, GNAQ, and GNA11 genes, establishing their neoplastic nature [68]. Various vascular tumors, from benign to malignant, have also been found to carry specific genetic aberrations that aid in their diagnosis and classification [69]. Mutations in PIK3CA, TEK, GNAQ, and GNA11 have been identified in various types of vascular malformations and hemangiomas [70]. Additionally, gene fusions involving FOS, FOSB, YAP1, and WWTR1 have been discovered in vascular tumors, improving diagnostic accuracy [71]. These genetic findings enhance our ability to diagnose and classify vascular neoplasms and provide insights into potential therapeutic targets and the underlying molecular pathways involved in their development.

### 4.8. Summary of the Above Findings (Table 4)

We summary the previous sections (from Section 4.1, Section 4.2, Section 4.3, Section 4.4, Section 4.5, Section 4.6 and Section 4.7) in Table 4. 

**Table 4:** Summary of the previous literature findings.

Variables	Main Findings
General characteristics of presentation of vulvar hemangiomas	Typically appear as bluish, red, or purple nodular or soft masses on the labia majora or vulvar region It may be painful or asymptomatic; pain may worsen during menstruation or with pressure It can present as cyclic perineal discomfort, swelling, or genital ulcers Lesions are often compressible and non-pulsatile, mimicking varicosities It may cause sexual dysfunction, cosmetic concerns, or psychological distress Occasionally associated with bleeding, ulceration, or secondary infection in larger or traumatized lesions
Clinical presentation and symptoms	Visible vulvar mass or nodules, often bluish or purplish Pain or tenderness, which may be intermittent or cyclic (worsening with menstruation) Swelling or enlargement of the lesion, especially during hormonal fluctuations Genital ulcers or skin breakdown over the lesion, in some cases Dyspareunia (pain during intercourse) or apareunia (inability to have intercourse) Bleeding from the lesion, particularly if traumatized or ulcerated Cosmetic concerns due to lesion appearance Possible psychological impact, including anxiety or distress related to symptoms or appearance
Image diagnosis of vulvar hemangiomas	Color Doppler ultrasonography: first-line imaging to detect tortuous vessels and turbulent blood flow, confirming vascular nature Transvaginal ultrasound: useful for assessing deep or pelvic extension of vulvar masses MRI characteristics: hemangiomas typically show hyperintense signals on T2-weighted images and contrast enhancement
Immunohistochemical characteristics	CD31: strongly positive; a sensitive marker for endothelial cells CD34: positive in the endothelial lining; helps confirm the vascular origin FLI-1: positive nuclear staining; supports the diagnosis of vascular tumors
New perspectives on therapy	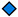 Conservative Management Observation may be appropriate for asymptomatic or stable lesions, given the potential for spontaneous regression This is particularly relevant in cases without significant pain, bleeding, or cosmetic/functional concern 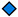 Medical Therapy Beta-blockers (e.g., propranolol, timolol) are effective, especially for infantile hemangiomas Oral propranolol (2 mg/kg/day) is more effective than topical timolol Corticosteroids are used when beta-blockers are contraindicated or ineffective Sclerotherapy offers a minimally invasive option 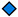 Minimally Invasive Interventions Laser therapy (e.g., CO_2_ laser, photodynamic therapy) is effective for certain superficial lesions Cryotherapy is promising for superficial or lymphatic lesions, showing lower recurrence rates than laser 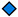 Surgical Excision Remains the mainstay for symptomatic or refractory cases Offers both diagnostic confirmation and definitive treatment Complete resection can provide durable relief but may not be preferred by all patients
Research gaps and future directions	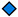 Need for Standardized Treatment Guidelines No current evidence-based guidelines for managing vulvar hemangiomas Treatment decisions vary widely, often based on clinician experience, not standardized protocols A multidisciplinary approach (gynecologists, dermatologists, vascular surgeons, radiologists) is essential 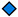 Advances in Targeted Therapies Propranolol-loaded nanoparticles and CD133-targeted systems offer sustained, selective inhibition of hemangioma stem cells Low-dose rapamycin suppresses endothelial proliferation via the mTOR/VEGF pathway Computational studies have identified drugs (e.g., sunitinib, sorafenib, dasatinib) that inhibit tumor growth and angiogenesis 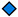 Role of Genetic and Molecular Studies Genetic mutations (e.g., GNA14, GNAQ, GNA11, PIK3CA, TEK) help confirm the neoplastic nature of hemangiomas Gene fusions (e.g., FOS, FOSB, YAP1) improve diagnostic accuracy and classification of vascular tumors

### 4.9. Comparison with One Previous Study

We compared the current study with one previously published one [16] and the summary in Table 5.

### 4.10. Strengths and Weaknesses

This review on vulvar hemangioma demonstrates several strengths. A comprehensive search strategy was employed, resulting in the initial identification of 136 articles, with duplicates systematically removed to ensure accuracy. Two independent reviewers conducted the screening process, enhancing reliability and minimizing potential selection bias. After applying predefined inclusion and exclusion criteria, 85 studies were included, reflecting a consistent and transparent selection process.

However, some limitations should be noted. The absence of exclusions during full-text review may suggest that the exclusion criteria were not sufficiently stringent or clearly defined. Additionally, the reported numbers contain a potential discrepancy, which could affect the transparency of the selection process. Lastly, given the rarity of vulvar hemangioma, the included studies likely consist mainly of case reports and series, introducing a risk of publication bias toward more severe or unusual cases.

## 5. Conclusions

A literature review of previous cases shows that vulvar hemangiomas affect patients of all ages and present with varying symptoms, including swelling, bleeding, pain, or sexual dysfunction. However, the treatment approaches remain inconsistent. Treatment should be symptom-driven, with asymptomatic cases monitored and symptomatic cases treated with surgical excision, embolization, or medical therapy as needed. Management by a multidisciplinary team involving gynecologists, dermatologists, vascular surgeons, and radiologists is crucial for optimal care. Given the wide variations in treatment strategies, the current case underscores the need for standardized guidelines to ensure consistent diagnosis, monitoring, and evidence-based management of vulvar hemangiomas.

## Figures and Tables

**Table 1:** Search strategy.

Items	Specification
Timeframe	From inception to 20 February 2025
Database	PubMed, Scopus, Web of Science, and Embase
Search term used	“vulvar hemangioma”
Inclusion and exclusion criteria	SCI-indexed articles written in English. Reviews, editorials, and studies unrelated to vulvar hemangiomas were excluded.
Selection process	Two independent reviewers evaluate the titles and abstracts to determine eligibility.

**Table 2:** Summary of previously reported cases.

Author, Year of Publication	Age (Years)	Parity	Past Medical History	Signs and Symptoms	Lesion Size	Treatment	Pathology/ Diagnosis
Abreu-Dos-Santos et al., 2016 [7]	51	3	Hypertension and hyperthyroidism	It looks like a small wart	2 cm long	Wide excision	Vulvar hemangioma
Besta et al., 2008 [8]	40	NA	Cervical spondylosis, right torticollis, capsulitis of the right shoulder, and seizure	Recurrent non-painful vulval lumps	2 cm	Biopsy	Cavernous hemangioma
Beva et al., 2002 [9]	1	NA	NA	Significant local bleeding caused by deep ulcerations	NA	Embolization	Immature capillary hemangioma
Bruni et al., 2009 [10]	20	NA	Mild allergic asthma, oligomenorrhea	Clitoromegaly	5 × 3 cm	Excision	Cavernous hemangioma
Cebesoy et al., 2008 [1]	26	NA	NA	Sexual dysfunction, pain, pressure	Covering the labia major	Excision	Cavernous hemangioma
Cheung et al., 2018 [3]	63	NA	NA	Discomfort and multiple purple-blue swellings were noted	Involving almost the whole of the right labia majora	No treatment	Cavernous hemangioma
Djunic et al., 2009 [11]	33	Preg 26 weeks	NA	Low-grade disseminated intravascular coagulation during pregnancy	Involving the left leg, including the foot, lower leg, and femoral region, as well as the left gluteal region and the left labia major and minor	Low-molecular-weight heparin	Cavernous hemangioma
Madhu et al., 2011 [12]	13	NA	Left breast cyst post cystectomy	Vulva swelling	1 × 1 cm	Excision	Benign hemangiomata
Mondal et al., 2017 [13]	42	NA	NA	Rapidly growing, glistening, ulcerative, pedunculated vulvar mass	NA	Excision	Lobular capillary hemangioma
Nayyar et al., 2014 [14]	10	NA	Her family history was not significant, and there was no history of any medication	Clitoromegaly	6 × 2.5 cm	Biopsy	Cavernous hemangioma
Peter et al., 2024 [15]	5	NA	Klippel–Trénaunay syndrome	Bleeding vulvar hemangioma	Located at left labia minora	Compression bandaging and timolol 0.2% solution	Hemangioma
Silva et al., 2018 [2]	52	NA	Prediabetes, dyslipidemia, and premenopausal	Genital ulcer for the past 3 years	Located at the perineal body	Creams and ointments, biopsy, surgical excision	Vulvar hemangioma
Current study	69	2	Type 2 diabetes mellitus and hypertension	Vulva nodules	Two small nodules	No treatment	Cavernous hemangioma

NA: not available.

**Table 3:** Comparative histopathological and immunohistochemical features of vulvar hemangioma and its major mimickers.

Entity	Histopathological Features	Immunohistochemical Profile	Key Differential Points
Cavernous Hemangioma (typical vulvar type)	- Dilated, blood-filled vascular spaces - Lined by flat, bland endothelial cells - Located in dermis/subcutaneous tissue - May contain thrombosis or phleboliths	CD31+ CD34+ FLI-1+ GLUT-1− (in adults) D2-40−	Benign; compressible; lacks nuclear atypia or infiltrative borders
Lymphangioma [39]	- Dilated, endothelium-lined lymphatic channels - Contains proteinaceous fluid (not erythrocytes) - Often clustered in dermis - May have superficial epidermal changes	D2-40+ CD31+ PROX1+ ± CD34 GLUT-1−	Clear or yellowish vesicles Absence of blood-filled spaces Positive D2-40 helps differentiate
Angiokeratoma [40]	- Small, thin-walled vascular spaces in upper dermis - Overlying epidermal hyperkeratosis, acanthosis - Minimal endothelial proliferation	CD31+ CD34+ GLUT-1−	Typically smaller, superficial Black or purple “wart-like” lesions Marked epidermal changes
Aggressive Angiomyxoma [41]	- Hypocellular, myxoid stroma - Numerous thick-walled, often hyalinized vessels - Infiltrative growth pattern - Scattered spindle cells	Desmin+ ER/PR+ CD34+ (variable) ± SMA+ GLUT-1−	Deep, infiltrative mass Recurring locally Often ER/PR positive; not purely vascular
Kaposi Sarcoma (vascular malignancy) [42]	- Spindle cell proliferation - Slit-like vascular spaces - Extravasated RBCs, hemosiderin, mitoses - Plasma cell infiltrate	CD31+ CD34+ HHV-8+ ± D2-40	Malignant; shows atypia, mitoses HHV-8 positive is diagnostic
Angiosarcoma (vascular malignancy) [43]	- Irregular, anastomosing vascular channels - Cytologic atypia, high mitotic activity - Infiltrative and destructive pattern	CD31+ ERG+ CD34+ ± Factor VIII+ GLUT-1−	Highly aggressive Distorted architecture High nuclear grade and mitoses

**Table 5:** Compare our study with the previous study.

Aspect	Our Study	Pediatric Study Summary
Population Focus	Primarily adult women with vulvar hemangiomas	Pediatric patients, especially with vulvovaginal and uterine hemangiomas
Hemangioma Types	Includes cavernous, arteriovenous, and lobular capillary hemangiomas	Focuses on infantile hemangiomas (IHs) and congenital hemangiomas (CHs)
Anatomical Focus	Vulvar lesions, particularly in the labia majora	Cervicovaginal and uterine lesions are more frequently described
Diagnostic Modalities	Emphasizes clinical exam, Doppler ultrasound, MRI, and histopathology with IHC	Similar imaging tools; highlight vaginoscopy, cystoscopy, and GLUT-1 staining for IHs
Immunohistochemistry	Uses CD31, CD34, FLI-1; GLUT-1 typically negative in adult lesions	GLUT-1 positive is a key marker for infantile hemangiomas
Treatment Options	Comprehensive: observation, beta-blockers, corticosteroids, sclerotherapy, laser, cryotherapy, excision	Propranolol as first-line; corticosteroids second-line; surgery for refractory or large lesions

IHC: immunohistochemistry, MRI: magnetic resonance imaging.

## Data Availability

The original contributions presented in this study are included in the article. Further inquiries can be directed to the corresponding author.
